# Fabrication and Performance Study of 3D-Printed Ceramic-in-Gel Polymer Electrolytes

**DOI:** 10.3390/gels11070534

**Published:** 2025-07-10

**Authors:** Xiubing Yao, Wendong Qin, Qiankun Hun, Naiyao Mao, Junming Li, Xinghua Liang, Ying Long, Yifeng Guo

**Affiliations:** 1 Guangxi Key Laboratory of Automobile Components and Vehicle Technology, Guangxi University of Science & Technology, Liuzhou 545006, China; 18300084899@163.com (X.Y.); qinwendong@163.com (W.Q.); 13383371921@163.com (Q.H.); maonaiyao@qmail.com (N.M.); 2 Liuzhou Institute of Technology, Liuzhou 545616, China; longying@lzhit.edu.cn; 3 School of Mechanical Engineering, Chengdu University, Chengdu 610106, China; guobujia2000@163.com

**Keywords:** 3D printing, porous structure, lithium-ion batteries

## Abstract

Solid-state electrolytes (SSEs) have emerged as a promising solution for next-generation lithium-ion batteries due to their excellent safety and high energy density. However, their practical application is still hindered by critical challenges such as their low ionic conductivity and high interfacial resistance at room temperature. The innovative application of 3D printing in the field of electrochemistry, particularly in solid-state electrolytes, endows energy storage devices with attractive characteristics. In this study, ceramic-in-gel polymer electrolytes (GPEs) based on PVDF-HFP/PAN@LLZTO were fabricated using a direct ink writing (DIW) 3D printing technique. Under the optimal printing conditions (printing speed of 40 mm/s and fill density of 70%), the printed electrolyte exhibited a uniform and dense sponge-like porous structure, achieving a high ionic conductivity of 5.77 × 10^−4^ S·cm^−1^, which effectively facilitated lithium-ion transport. A structural analysis indicated that the LLZTO fillers were uniformly dispersed within the polymer matrix, significantly enhancing the electrochemical stability of the electrolyte. When applied in a LiFePO_4_|GPEs|Li cell configuration, the electrolyte delivered excellent electrochemical performance, with high initial discharge capacities of 168 mAh·g^−1^ at 0.1 C and 166 mAh·g^−1^ at 0.2 C, and retained 92.8% of its capacity after 100 cycles at 0.2 C. This work demonstrates the great potential of 3D printing technology in fabricating high-performance GPEs. It provides a novel strategy for the structural design and industrial scalability of lithium-ion batteries.

## 1. Introduction

With the rapid development of electric vehicles, portable electronic devices, and renewable energy sources, the demand for energy storage devices with high energy density, strong safety, and long life cycles is increasing. Solid-state lithium-ion batteries (SSLIBs), which utilize solid-state electrolytes instead of traditional liquid electrolytes, offer improved thermal stability and safety and have attracted increasing attention as next-generation energy storage devices [1,2]. However, current all-solid-state batteries still face multiple challenges regarding their ionic conductivity, interfacial stability, and manufacturing processes, making large-scale commercialization difficult [3]. Gel polymer electrolytes (GPEs), combining the structural integrity of solid-state systems with the ionic conductivity of liquid-like phases, provide a promising intermediate solution. GPEs typically consist of a polymer matrix, lithium salt, and a small amount of plasticizer or solvent retained within the polymer network, forming a quasi-solid-state phase that enhances ion transport and electrode interfacial compatibility [4]. This class of materials maintains mechanical flexibility while exhibiting high ionic conductivity, making them attractive for safe and efficient lithium-ion batteries.

There are various preparation methods for gel electrolytes, such as solution casting [5], the Phase Inversion Method [6], and electrospinning technology [7]. Although these traditional processes are mature, they still have limitations in terms of uniformity, structure controllability, and interface design. With its flexible processing and precise structural control capabilities, 3D printing technology has gradually been applied to the fabrication of multifunctional and shape-customized gel polymer electrolytes [8].

Poly(vinylidene fluoride)-hexafluoropropylene (PVDF-HFP) is a copolymer based on crystalline -VDF and amorphous -HFP segments [9]. PVDF-HFP is widely studied due to its low crystallinity and glass transition temperature (−62 °C). Its repeating unit, -CH_2_-CF_2_-, has a strong electron-withdrawing group and exhibits high polarity [10]. PVDF-HFP also possesses excellent chemical stability, excellent mechanical properties, and a high dielectric constant [11]; its amorphous regions help improve ionic conductivity [12]. However, a single polymer system can no longer meet the growing demands for modern dielectric materials [13]. Polyacrylonitrile (PAN), due to its strong polar electron-withdrawing nitrile group, C≡N, has strong molecular interactions with lithium salts and thus exhibits high ionic conductivity [14]. It also performs well in terms of its thermal stability and interfacial oxidation [15]. Notably, PVDF-HFP has excellent ionic conductivity, high elongation at break, and good miscibility with other polymers [16]. A composite system of PVDF-HFP and PAN can thus effectively balance ionic transport and mechanical robustness. To further improve the performance of GPEs, ceramic fillers such as garnet-type Li_6.4_La_3_Zr_1.4_Ta_0.6_O_12_ (LLZTO) are commonly introduced. LLZTO provides high intrinsic ionic conductivity and enhances the structural stability of the composite electrolyte [17]. The resulting composite structure, combining organic polymers with inorganic ceramic fillers, exhibits both gel-like ionic conduction pathways and solid-state mechanical strength.

In the early stage of applying 3D printing to polymer electrolytes, most studies focused on electrochemical performance. Aaron J. Blake [18] established a method for 3D printing porous electrolytes using a dry-phase conversion technique. These electrolytes demonstrated similar high-rate performance (e.g., 5 C) to commercial polyolefin separators but had better wettability and thermal stability. Meng Cheng [19] proposed a new approach to fabricate hybrid solid-state electrolytes using high-temperature direct ink writing (DIW) without solvent evaporation. This method improved both the capacity and rate performance. Later, Zixian Liu [20] developed slurries based on lithium aluminum titanium phosphate (LATP), poly(ethylene oxide) (PEO), and lithium bis(trifluoromethanesulfonyl)imide (LiTFSI), exhibiting favorable rheological properties for direct ink writing (DIW). The printed solid-state electrolytes, applied directly onto LiFePO_4_ cathodes, achieved a high discharge capacity of 150 mAh g^−1^ at 0.5 C. Despite the promising electrochemical performance of LATP-based ceramics, their long-term cycling stability remains a challenge. Bo Chen [21] developed three LLZTO slurry formulations that enabled the printing of complex 3D battery structures with 150 μm resolution. These slurries had a high modulus, high yield stress, and clear shear-thinning behavior. Changgang Li [22] prepared PEO-LATP polymer electrolytes using 3D printing and introduced various lithium salts to stabilize the electrode/electrolyte interfaces, suppressing LiNi_0.8_Co_0.1_Mn_0.1_O_2_ (NCM811) degradation and dendrite formation. The printed solid-state batteries could cycle 4000 times at 2 C. Currently, 3D printing research on solid-state electrolytes remains focused on semi-solid systems (e.g., PEO-based), with limited studies on the systematic control of process parameters or in situ solidification strategies in non-PEO systems. These gaps hinder the practical development of high-safety, all-climate solid-state batteries.

In this work, we used DIW to design and fabricate 3D-printed gel polymer electrolytes. PVDF-HFP/PAN@LLZTO inks were prepared, their rheology was measured, and the printing parameters were optimized. Physical and electrochemical properties were compared across different thicknesses. The porous structure provided continuous channels for lithium-ion transport. The 90 μm thick electrolyte showed the highest ionic conductivity and excellent electrochemical performance. This research provides a new method for enhancing GPEs’ performance and exploring novel paths for all-solid-state battery development.

## 2. Results and Discussion

**Figure 1:**
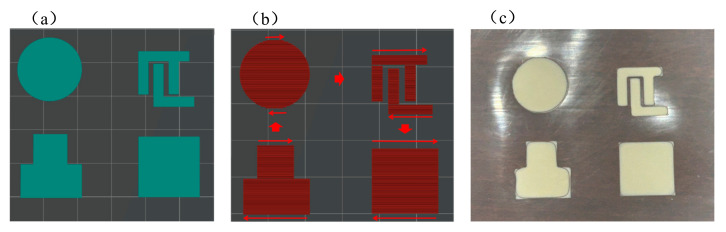
GPEs with customized shapes: (**a**) schematic; (**b**) printing routine; (**c**) macroscopic image.

In specific applications, batteries often need to be customized to different geometries under different conditions. Three-dimensional printing provides an effective solution to this technical challenge by enabling the fabrication of gel polymer electrolytes with customized geometries through simple adjustments to the printing program. As shown in Figure 1a–c, under consistent process parameters, we printed circular, maze-shaped, convex, and square GPEs. Through 3D printing technology, efficient material utilization, customizable structural design, and functional integration can be achieved, providing a new perspective for improving gel polymer electrolyte performance.

**Figure 2:**
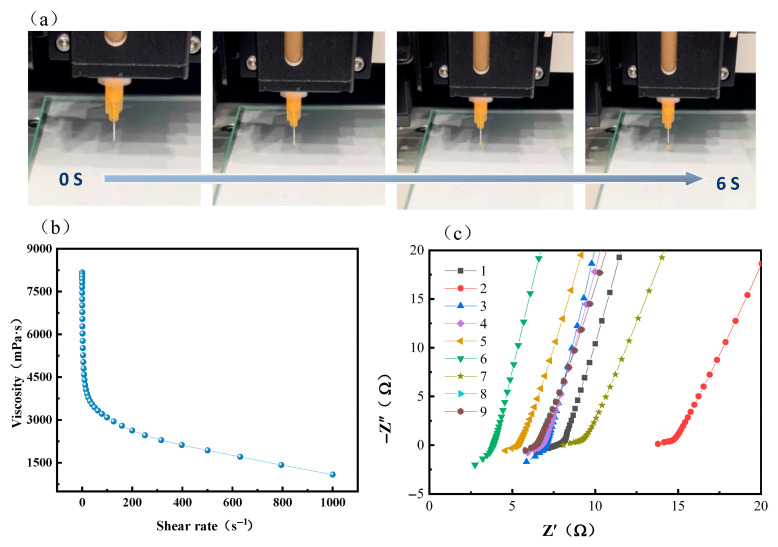
(**a**) Extrusion of slurry from syringe; (**b**) apparent viscosity of slurry as function of shear rate value; (**c**) Nyquist plots of different process parameters.

Ink with good rheological properties is the key to being able to perform continuous 3D printing, and observing the flow state of the paste ink during extrusion is one of the indicators for evaluating the rheological properties [20]. The prepared ink was loaded into a syringe and the continuous extrusion ability of the ink was observed, as shown in Figure 2a. Under a pressure of 0.06 MPa, continuous ink extrusion could be observed within 6 s. The viscosity of the paste has a great influence on the rheology within the coating gap, as well as the quality of the wet film, and with an increase in the shear rate, the electrolyte ink exhibits shear-thinning characteristics. During the printing process, the shear-thinning property favors the continuous extrusion of the slurry when the coating gap is subjected to high shear, while the near-zero shear viscosity, which is elevated when the shear is very low, ensures good stability of the shape of the wet film aggregate during the drying process [23]. Figure 2b shows the rheological properties of the slurry, which can be seen to exhibit shear thinning, which is essential for successful printing. To determine the optimal process parameters, nine sets of experiments were conducted with the printing speed and infill density as variables. The printing speed was set to 20, 40, or 60 mm/s, and the infill density was selected as 50%, 70%, or 90%. The 3D printing process was then optimized based on the ionic conductivity results, as summarized in Table 1. As shown in Figure 2c, the bulk resistance obtained from Nyquist plots indicates that the optimal printing parameters are a speed of 40 mm/s and a filling density of 70%, corresponding to the highest ionic conductivity (Sample No. 5, 5.77 × 10^−4^ S·cm^−1^) calculated using Equation (1).

**Figure 3:**
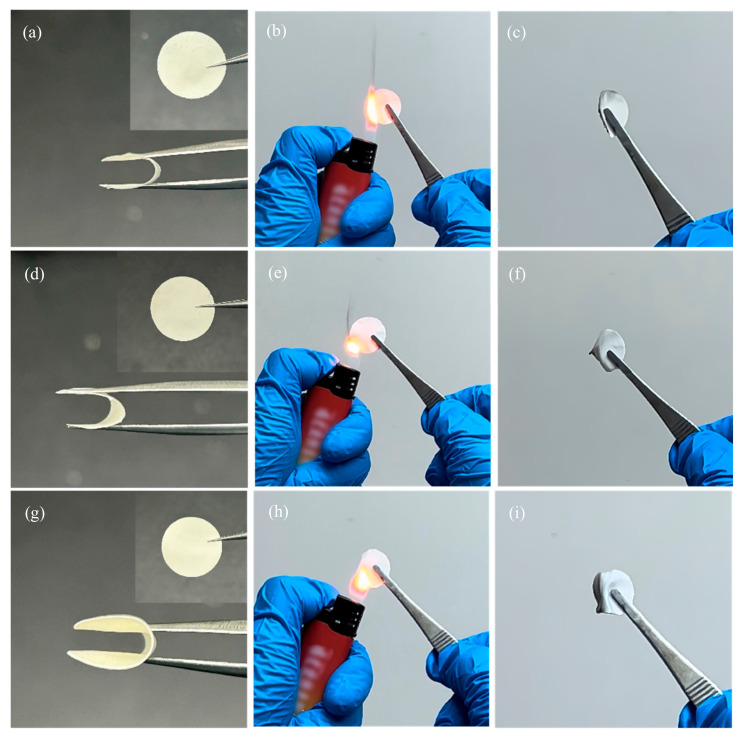
(**a**–**c**) Front and folded views, ignition moment, and morphology after flame removal of GPE_50; (**d**–**f**) front and folded views, ignition moment, and morphology after flame removal of GPE_90; (**g**–**i**) front and folded views, ignition moment, and morphology after flame removal of GPE_130.

In order to investigate the effect of different thicknesses on the electrolyte, three different thicknesses of electrolyte films, 50 (±10) μm, 90 (±10) μm, and 130 (±10) μm, were prepared by controlling the air pressure during printing. As can be seen from Figure 3a–c, the prepared polymer electrolyte films were white in color with a flat and smooth surface; could recover their original state when arbitrarily bent, coiled, or folded; and had good plasticity and flexibility. To evaluate the flame-retardant properties of the GPEs, vertical combustion tests were conducted. As shown in Figure 3b,c, GPE_50, with its loosely porous internal structure, readily ignited upon exposure to a flame. Once the flame was removed, it underwent significant shrinkage and deformation. In contrast, as depicted in Figure 3e,f,h,i, GPE_90 and GPE_130, due to their relatively denser porous structures and greater thicknesses, exhibited reduced flammability when exposed to a flame. Moreover, they retained more intact morphologies after the flame was removed, demonstrating improved flame-retardant properties.

**Figure 4:**
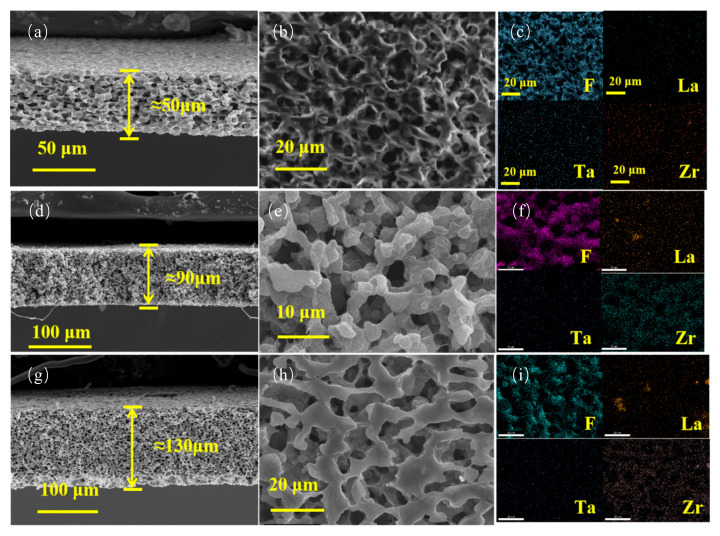
(**a**–**c**) Cross-sectional SEM, surface SEM, and EDS maps of GPE_50; (**d**–**f**) cross-sectional SEM, surface SEM, and EDS maps of GPE_90; (**g**–**i**) cross-sectional SEM, surface SEM, and EDS maps of GPE_130.

The structural characteristics of electrolyte membranes with different thicknesses were investigated, as shown in Figure 4. All samples exhibited uniform sponge-like porous structures, which were attributed to the breath figure method. During the natural drying process of the freshly printed wet membrane in ambient air, solvent evaporation leads to a localized temperature drop, causing water droplets to condense on the surface. Polymer chains accumulate around these droplets, forming polymer shells. As drying progresses, the water droplets undergo orderly rearrangement under the influence of surface capillary forces, eventually organizing into regular hexagonal arrays, leaving behind a porous structure after evaporation [24]. This porous morphology is advantageous for constructing continuous lithium-ion transport channels. In particular, the presence of larger pores enhances the interfacial contact area between the electrolyte and electrode, facilitating fast ion diffusion and efficient charge transfer kinetics [25]. As shown in Figure 4a,b, GPE_50 exhibited a fluffy and uniform sponge-like porous structure. The Energy Dispersive X-ray Spectroscopy (EDS) mapping in Figure 4c confirmed the homogeneous distribution of F, La, Ta, and Zr elements without visible aggregation, suggesting good dispersion of LLZTO fillers within the polymer matrix. Figure 4d,e reveal that the GPE_90 membrane presented a denser sponge-like structure, while its corresponding EDS mapping (Figure 4f) further confirmed uniform elemental distribution, again suggesting effective filler dispersion. Figure 4g,h demonstrate that GPE_130 exhibited a dense sponge-like structure; however, Figure 4i reveals partial agglomeration of La elements, which may have resulted from excessively high printing pressure disrupting the ink’s homogeneity and causing filler aggregation. Overall, GPE_90, fabricated under optimal printing pressure, demonstrated balanced porosity and high compactness, contributing to efficient and uniform lithium-ion transport while maintaining strong mechanical integrity. In addition, the small and uniform particle size of LLZTO fillers facilitates their homogeneous dispersion in the polymer matrix, which is beneficial for forming continuous Li^+^ transport pathways and improving electrochemical performance [26]. The uniform distribution of fillers is critical for enhancing both the electrochemical performance and thermal stability of the electrolyte [27]. Notably, Figure 4f shows that the F element was more evenly distributed and more broadly covered in GPE_90, suggesting that PVDF-HFP was better dispersed in this sample, leading to more effective integration between the polymer and ceramic fillers. This further highlights the morphological and structural advantages of the GPE_90 membrane.

**Figure 5:**
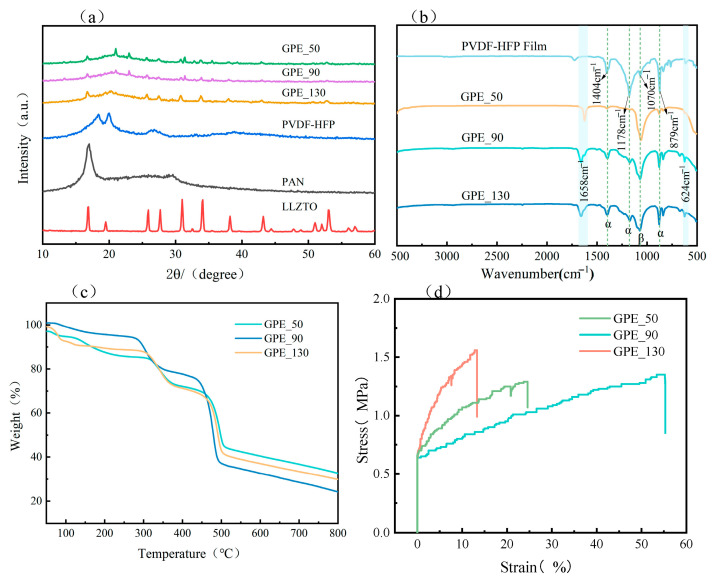
(**a**) XRD patterns of GPEs with different thicknesses; (**b**) FTIR spectra of the pure PVDF-HFP membrane and GPEs with different thicknesses; (**c**) thermogravimetric analysis of GPE membranes; (**d**) stress–strain curves of GPEs with different thicknesses.

To further illustrate the amorphous nature of the GPEs, an X-ray diffraction (XRD) analysis was performed. Figure 5a shows the XRD patterns of LLZTO, PAN, PVDF-HFP, GPE_50, GPE_90, and GPE_130. The results show that the characteristic peaks of LLZTO remained clearly observable in the electrolyte, and the main diffraction peaks of pure PVDF-HFP were observed at 2θ = 18.2°, 20.0°, and 26.6°, which correspond to the planes of semicrystalline polymers (100), (020), and (110), respectively. After blending, the characteristic peaks of PVDF-HFP were weakened, indicating that the PVDF-HFP had reduced crystallinity, which may have resulted from molecular interactions with PAN and the lithium salt. A slight shift of the (020) diffraction peak of PVDF-HFP to lower 2θ angles was observed after blending, which can be attributed to the interaction between PVDF-HFP and PAN chain segments, as well as the Lewis acid–base interaction between LLZTO and the polymer matrix [28]. The diffraction peaks of LLZTO stabilized in the electrolyte, and the crystal structure of LLZTO did not change, which indicates that LLZTO retained its structural integrity and electrochemical compatibility within the electrolyte film.

FTIR spectroscopy was employed to investigate the molecular interactions among the polymer matrix, LiClO_4_, and LLZTO in the GPEs, as shown in Figure 5b. The peak at 1658 cm^−1^ corresponds to C=O stretching in PAN, and the peak at 624 cm^−1^ corresponds to the presence of ClO_4_^−^ anions in the complexes, indicating coordination between the polymer and lithium salt [29]. In comparison with pure PVDF-HFP membranes, the α-phase peaks at 1404 cm^−1^ (C-H bending in CH_2_), 1178 cm^−1^ (-CF_2_ symmetric stretching) [30], and 879 cm^−1^ (C-C backbone vibration) were reduced in intensity in each GPE, while the β-phase (1070 cm^−1^, C-C stretching) [31] was enhanced. This could be attributed to the LLZTO filler in the GPEs promoting the enhancement of the β-phase, which facilitates the dissociation and migration of the lithium salt ions and improves the ionic conductivity. The decrease in the peak intensities at 1178 cm^−1^ and 1404 cm^−1^ suggests the dehydrochlorination of the PVDF-HFP-HFP strands, which could be attributed to the fact that the LLZTO filler plays the role of a Lewis base, inducing partial chemical dehydrofluorination, thus enhancing the interactions among the polymer matrix, lithium salt, and LLZTO particles [32].

The TG curves of the GPEs (Figure 5c) show three main weight loss stages. Below 300 °C is the decomposition of the residual solvent in the electrolyte and water absorbed from the air; from 300 °C is the second stage of thermal decomposition of PAN; and from 400 °C is the third stage, the decomposition of PVDF-HFP. It was previously reported that pure PVDF-HFP can remain stable without decomposition at 450 °C, while the GPEs slightly decomposed before reaching this temperature. This may be due to the addition of LiClO_4_ and LLZTO reducing their crystallinity [25], which is the same as the XRD results. As the temperature increased, at 800 °C, GPE_50 had the lowest residue (24%), while GPE_90 had the highest residue (32%). This may be attributed to the dense porous structure within GPE_90, which enhances the thermal stability of the electrolyte [33]. In a sense, the thermodynamic properties of the prepared gel polymer electrolyte membranes can meet the requirements of most solid-state lithium-metal battery applications.

The mechanical stability of the GPEs is a pivotal factor in maintaining the structural stability of a lithium-ion battery during cycling. As depicted in Figure 5d, GPE_130 exhibited the highest tensile strength of 1.5 MPa with an elongation at break of 15%, while GPE_90 demonstrated a moderate tensile strength of 1.2 MPa along with a significantly higher elongation of 55%. This mechanical behavior plays a crucial role in suppressing lithium dendrite growth during cycling.

**Figure 6:**
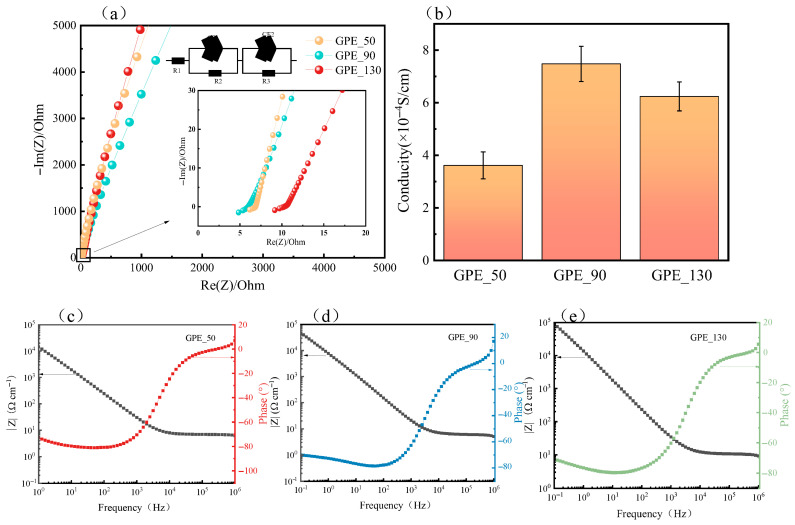
Electrochemical impedance spectroscopy (EIS) results for different GPE samples: (**a**) Nyquist plots for various GPEs; (**b**) ionic conductivity of GPE_90, with error bars indicating standard deviations; (**c**) Bode plot for GPE_50; (**d**) Bode plot for GPE_130; (**e**) Bode plot for GPE_90.

Ionic conductivity is an important indicator of electrochemical performance, and the ionic conductivity associated with different printing parameters is shown in Figure 6a. The bulk resistances of GPE_50, GPE_90, and GPE_130 were 6.877, 5.988, and 10.37 Ω, respectively, and according to Equation (1), the ionic conductivities were calculated to be 3.62 × 10^−4^, 7.48 × 10^−4^, and 6.24 × 10^−4^ S cm^−1^ (Figure 6b). These values demonstrate that the obtained GPEs, particularly GPE_90, exhibited relatively high ionic conductivity. For comparison, previous studies have reported ionic conductivity values of 4.24 × 10^−4^ S cm^−1^ and 7.4 × 10^−4^ S cm^−1^ for PVDF-HFP-based gel electrolytes under similar conditions [34,35], confirming the validity of our results. The enhanced performance of GPE_90 can be attributed to the homogeneous distribution of LLZTO fillers within the polymer matrix, which facilitates the formation of continuous lithium-ion conductive pathways [36]. In addition, the moderate printing pressure used in preparing GPE_90 results in a well-developed porous structure, which further supports efficient ion transport. Therefore, GPE_90 achieved the highest ionic conductivity among the tested samples. The equivalent circuit model used for fitting the impedance spectra included R1 representing the electrolyte resistance, R2 and R3 as the electrolyte–electrode interface resistances, and CE1 and CE2 as constant phase elements accounting for the non-ideal capacitive behavior. The simulated impedance response was directly calculated using ZView 3.1 software, and the good agreement between the experimental and simulated curves supports the validity of the fitted model. The Bode plots (Figure 6b–d) further confirmed that GPE_90 had low impedance and the highest phase angle across the frequency range, suggesting superior ion transport and interfacial behavior.

**Figure 7:**
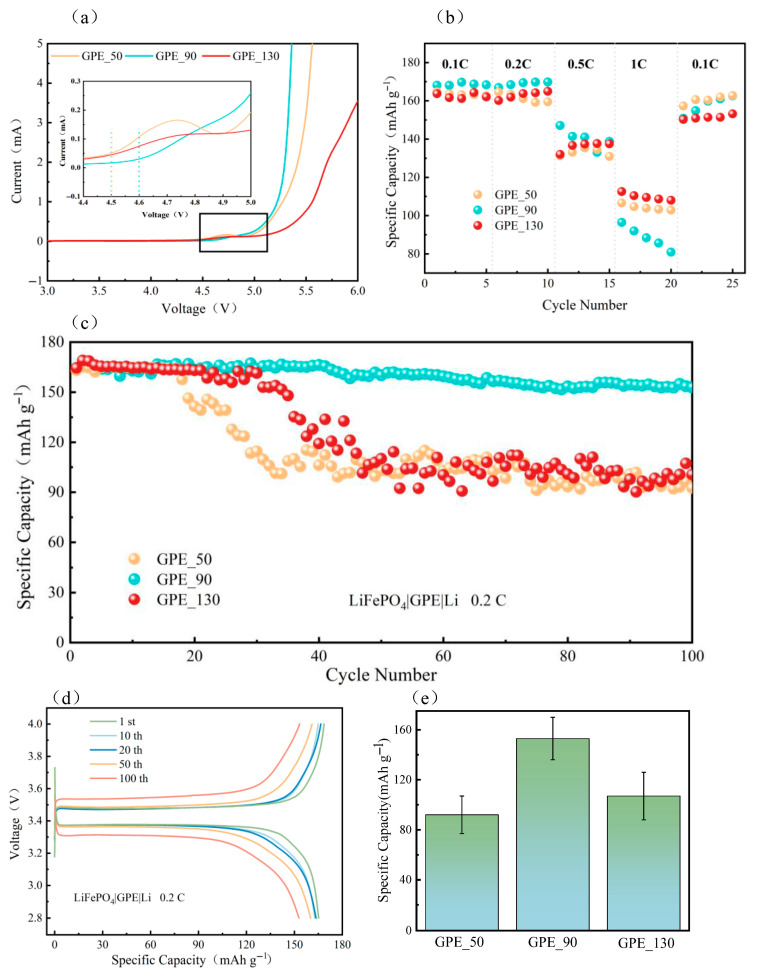
(**a**) Voltammetric linear scan curves of solid electrolytes with different thicknesses; (**b**) GPEs’ multiplicity performance for different thicknesses; (**c**) cycling plots of GPEs with different thicknesses; (**d**) charge/discharge curves of GPE_90 with different numbers of cycling turns; (**e**) residual capacity after cycling, with error bars indicating standard deviations.

Electrochemical stability is the basis for matching high-voltage electrodes. Linear scanning voltammetric curves were used to evaluate the oxidative stability of the electrolyte films. The ionic conductivities associated with different printing parameters are shown in Figure 7c. GPE_90 had a higher decomposition voltage (4.6 V), and GPE_50 and GPE_130 had similar electrochemical windows (4.5 V), which shows that there was no significant difference among the 3D-printed electrolytes. Further, there is a stabilization window below 4.5 V vs. Li/Li^+^, which suggests that they are able to match Li-FePhosphate batteries, and oxidative decomposition does not occur.

The multiplicity performance of LiFePO_4_| GPE| Li batteries at room temperature (25 °C) is shown in Figure 7c. It can be seen that the prepared GPEs all achieved a high specific capacity of discharge (above 160 mA·g^−1^) at low multiplicity, demonstrating good multiplicity performance. GPE_90 exhibited a higher specific capacity of discharge due to its higher ionic conductivity (the initial specific capacity of discharge can be up to 168 mAh·g^−1^), and GPE_130, due to its excessive thickness, led to a cell performance decrease, discharging the least specific capacity at low multiplicity. However, with an increase in current density, the growth of lithium dendrites was promoted, the internal side reaction of GPE_90 increased, and the battery exhibited unstable charging and discharging states with fast decay. Meanwhile, the GPE_50 discharge specific capacity decreased relatively slowly, which may be due to the lesser thickness shortening the ion transport path and reducing the ion transport resistance. Table 2 shows a performance comparison between the GPE films in this study and the existing solid-state electrolytes. It can be seen that the electrochemical performance of 3D printing-based solid-state electrolytes still needs to be further improved.

**Figure 8:**
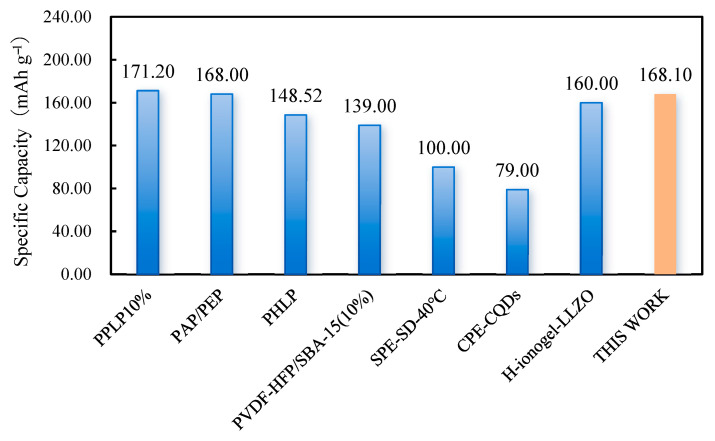
Comparison of discharge performance of existing solid-state electrolytes at 0.1 C multiplication rate.

The GPEs with different thicknesses were cycled for 100 cycles at 0.2 C magnification, and the results are shown in Figure 7c,e. The results show that GPE_90 was chemically stable at low magnification, and its capacity was maintained at 152.8 mAh g^−1^ after 100 cycles, with a capacity retention rate of 92.8%. The capacity of GPE_50 and GPE_130 decayed faster, with capacity retention rates of 56.7% and 61.1%, respectively, after 100 cycles. The side reaction between residual DMF and lithium metal during the cycling process resulted in a faster capacity decay of the battery. The thicker GPE_130 showed significant capacity decay after 30 cycles, which could be attributed to the fact that the thicker electrolyte reduced the risk of lithium dendrite puncture, but due to its internal interlayer structural defects, it led to the capacity decay of the battery. On the other hand, GPE_90 exhibited higher discharge capacity and lower capacity decay with stronger cycling stability and dendrite resistance due to the moderate printing air pressure, which formed a good internal structure and balance between continuous Li^+^ channels and dendrite resistance. The charging/discharging curves (Figure 7d) under different cycles corresponding to the performance of the GPE_90 cell at 0.2 C at room temperature (25 °C) show that the voltage plateaus in the first 50 cycles tended to overlap (the polarization voltage was 0.1 V) with small differences, indicating the reversibility and stability of GPE_90 in long-time operation [37]. The polarization voltage of charge/discharge reached 0.2 V at the end of the 100-lap cycle, which may be due to the deterioration of the electrode–electrolyte interface contact due to the volume change and interfacial side reaction during the cycling process, which increases the interfacial resistance and leads to an increase in the polarization voltage. In addition, lithium dendrite formation is a critical challenge impacting the long-term cycling stability and safety of lithium-metal batteries. The enhanced cycling performance and dendrite suppression observed in GPE_90 can be attributed to its optimized internal microstructure achieved through the 3D printing process. This structure promotes uniform Li^+^ ion flux, effectively inhibiting the nucleation and growth of lithium dendrites, thereby reducing the risk of short circuits and capacity fading under moderate current densities. However, at elevated current densities, the accelerated growth of lithium dendrites and intensified side reactions contribute to the observed faster capacity decay [38,39]. Figure 8 demonstrates a comparison of the discharge performance of different processes and material systems at 0.1 C multiplication.

**Table 2:** Performance comparison of existing solid-state electrolytes.

SSEs	Method	0.1 C (mAh g^−1^)	0.2 C (mAh g^−1^)	0.5 C (mAh g^−1^)	After Cycles (mAh g^−1^)	Ref.
PPLP10%	Solution casting	171.20 (25 °C)	\	167.20 (25 °C)	157.30, 97.8% (1 C, 100)	[40]
PAP/PEP	Solution casting	168.00 (25 °C)	150.00 (25 °C)	120.40 (25 °C)	140.00, 93.3% (0.2 C, 100)	[29]
PHLP	Solution casting	148.52 (25 °C)	141.74 (25 °C)	118.68 (25 °C)	123.16, 88.29% (0.33 C, 200)	[41]
LLZO/LIC LISE	In situ sintering	167.20 (60 °C)	165.50 (60 °C)	\	144.20, 89.0% (0.5 C, 200)	[42]
PVDF-HFP/SBA-15 (10%)	3D printing	139.00 (25 °C)	\	\	\	[43]
LiPF6 Gel10	3D printing	\	145.00 (25 °C)	144 (25 °C)	117.00, 96% (1 C, 150)	[44]
SPE-SD-40 °C	3D printing	100.00 (40 °C)	40.00 (40 °C)	\	\	[45]
GPE-CQDs	3D printing	79.00 (25 °C)	78.00 (25 °C)	74.00 (25 °C)	74.00, 92% (0.2 C, 200)	[46]
H-ionogel-LLZO	3D printing	160.00 (50 °C)	153.00 (50 °C)	148.00 (50 °C)	125.00, 88.1% (0.5 C, 200)	[47]
This work (GPE_90)	3D printing	168.10 (25 °C)	166.90 (25 °C)	147.00 (25 °C)	152.80, 92.8% (0.2 C, 100)	This Work

## 3. Conclusions

In this study, PVDF-HFP/PAN@LLZTO gel polymer electrolytes were successfully prepared by 3D printing technology, and their rheological properties, physical properties, and electrochemical properties were systematically explored. It was shown that the optimized paste formulation and rheological properties ensured the controllability and stability of the 3D printing process, which enabled continuous extrusion and high-precision molding. The shear-thinning property enabled the electrolyte slurry to have good rheological behavior at high shear rates, while the appropriate near-zero shear viscosity ensured the stability of the wet film morphology during the drying process. The optimized 3D printing process parameters (printing speed of 40 mm/s, filling density of 70%) significantly enhanced the ionic conductivity of the solid electrolytes, among which the GPE_90 sample exhibited the highest ionic conductivity (7.48 × 10^−4^ S cm^−1^) and lower interfacial impedance. The SEM results showed that the electrolytes with different thicknesses exhibited a uniform sponge-like porous structure, and the LLZTO fillers were uniformly distributed in the polymer matrix, improving the lithium-ion transport efficiency. The thermogravimetric (TG) analysis results further verified the thermal stability of the GPEs, which can meet the application requirements of lithium-metal batteries. Additionally, the printed membranes exhibited adequate mechanical strength to meet the basic handling and assembly requirements of quasi-solid-state batteries. The GPEs had a wide electrochemical window (4.6 V), good multiplicity performance (0.1 C@168 mAh·g^−1^,0.2 C@166 mAh·g^−1^), and good cycling performance (92.8% capacity retention after 100 cycles). Furthermore, considering its industrial application potential, the DIW 3D printing process used here offers distinct advantages in fabricating customized electrolyte membranes with precise control over their structure and composition. Scaling this process for industrial manufacturing will require further optimization of the printing speed, slurry formulation, and post-processing steps to ensure reproducibility and throughput, which will be a focus of subsequent research efforts. In summary, this study demonstrated the feasibility and advantages of 3D printing technology in the preparation of customized GPEs, which provides new ideas and technical support for the development of high-performance quasi-solid-state lithium-metal batteries.

## 4. Materials and Methods

### 4.1. Materials

PVDF, PVDF-HFP (Mn = 600,000, Arkema, Colombes, France), poly(acrylonitrile) (PAN, Mw = 85,000, Shanghai, China), N,N-Dimethylformamide (DMF, ≥99.9%), lithium perchlorate (LiClO_4_) (99.99%, Aladdin Chemical Co., Shanghai, China), N-methyl pyrrolidone (NMP, ≥99.9%), Super-P (≥99.5%), and LiFePO_4_ (LFP, ≥99.5%) were all obtained from Krud in Shanghai, China. Lithium lanthanum zirconium tantalum oxide (LLZTO, average particle size ~500 nm) was also obtained from Krud.

### 4.2. Preparation of Gel Polymer Electrolytes (GPEs)

Preparation of ink: A certain amount of PVDF-HFP was weighed and dissolved in DMF on a magnetic stirrer at 45 °C. In order to obtain well-dispersed printable ink, PAN powder was mixed with the PVDF-HFP at a weight ratio of 7:3, and LLZTO (10 wt% relative to the total polymer mass) was thoroughly ground with the polymer blend using an agate mortar and pestle. After the PVDF-HFP was dissolved, the ground PAN and LLZTO powders were added. The polymer matrix and filler were stirred well, then LiClO_4_ (30% by mass to polymer) was added, stirred well for 4 h, and ultrasonicated for 30 min for defoaming.

**Figure 9:**
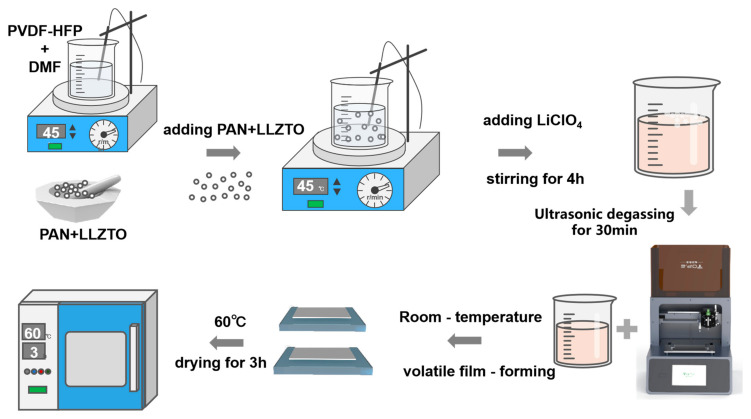
Preparation of gel polymer electrolyte membrane.

Preparation of GPEs: The preparation process of the composite polymer electrolyte is shown in Figure 9. Once the ink was prepared, it was loaded into a syringe equipped with an extrusion nozzle tip (inner diameter of 0.34 mm), and a 3D printer with pneumatically driven extrusion was used to perform the DIW process on quartz glass. In order to obtain the best printing parameters, nine sets of experiments were conducted to optimize the printing parameters, including the speed and filling density. The printed composite polymer electrolytes were placed in a drying oven and dried for 3 h, after which they were cut into 19 mm discs and placed in a glove box (H_2_O < 0.01 ppm, O_2_ < 0.01 ppm) to rest for 24 h before use.

Gel polymer electrolyte films with controlled nominal thicknesses of 50 ± 10 μm, 90 ± 10 μm, and 130 ± 10 μm (systematically designated GPE_50, GPE_90, and GPE_130) were fabricated through precise adjustment of the printing pressure while maintaining all other processing parameters consistent with the reference methodology.

### 4.3. Assembling the Battery

LiFePO_4_ (LFP) was used as the cathode and lithium metal as the anode for cell assembly. All components, including LFP electrodes, lithium foil, stainless steel spacers, and GPE membranes, were dried at 60 °C for 12 h prior to use. During assembly, approximately 10 μL of liquid electrolyte (1 M LiPF_6_ in EC/DMC/EMC = 1:1:1 Vol% with 10% FEC 2% VC) was drop-cast onto the surface of the GPEs to promote polymer swelling and improve interfacial contact. The assembled CR2025 coin cells were rested for 12 h before subsequent electrochemical characterization.

### 4.4. Material Characterization

Rheological properties critical to the printing performance of the pastes were measured using a rotational rheometer (MCR302, Anton Paar, Graz, Austria) equipped with a 27 mm diameter cylinder. The viscosity of the slurries was tested within a shear rate range of 0.1 to 1000 s^−1^ at 25 °C to evaluate their shear-thinning behavior and printability. The crystalline structures of the dried GPE membranes and raw materials (PVDF-HFP, PAN, and LLZTO) were analyzed by X-ray diffraction (XRD; DX-2700, Dandong, China) with Cu Kα radiation at 40 kV and 30 mA, using a scan rate of 0.2°/s across a 2θ range of 10–90°. The surface and cross-sectional morphologies of GPE_50, GPE_90, and GPE_130 were observed and analyzed using scanning electron microscopy (SEM; Phenom Spectrum G2, Shanghai, China) after the samples were fractured in liquid nitrogen and sputter-coated with gold. Elemental mapping was performed using energy-dispersive X-ray spectroscopy (EDS; Phenom Spectrum G2, Shanghai, China) to assess the dispersion of ceramic particles. Fourier transform infrared (FTIR; Spectrum 100, PerkinElmer, Shelton, CT, USA) spectroscopy was used to determine the chemical bonding of GPE_50, GPE_90, and GPE_130, in the range of 500–3500 cm^−1^ using the ATR mode. The thermal stability of the GPEs was investigated using thermogravimetric analysis (TGA; METTLER TOLEDO DSC 3+) in a nitrogen atmosphere with ceramic crucibles, over a temperature range of 50 °C to 800 °C at a heating rate of 10 °C min^−1^.

### 4.5. Electrochemical Properties

The GPEs were assembled into a symmetric cell structure of steel sheet (SS)/gel polymer electrolyte/steel sheet (SS) and tested for their electrochemical impedance spectra by performing AC impedance experiments at room temperature. The spectra were measured according to the equation

(1) $$\sigma =\frac{L}{R\cdot S}$$

where σ is the ionic conductivity (S·cm^−1^); L is the thickness of the electrolyte membrane (cm); R is the bulk impedance of the material (Ω); and S is the area of the gel polymer electrolyte (cm^2^). The electrolyte membrane was assembled into a lithium/gel polymer electrolyte/steel sheet battery structure for linear scanning (LSV) experiments with an initial potential of 2 V, a termination potential of 6 V, and a scanning rate of 5 mV/s.

## Figures and Tables

**Table 1:** Experiments with different parameter settings.

Number	Printing Speed (mm/s)	Filling Density (%)	Ionic Conductivity (S·cm^−1^)
1	20	50	2.9 × 10^−4^
2	20	70	1.98 × 10^−4^
3	20	90	3.18 × 10^−4^
4	40	50	2.8 × 10^−4^
5	40	70	5.77 × 10^−4^
6	40	90	2.93 × 10^−4^
7	60	50	1.09 × 10^−4^
8	60	70	9.41 × 10^−5^
9	60	90	1.47 × 10^−4^

## Data Availability

The original contributions presented in this study are included in the article. Further inquiries can be directed to the corresponding author.
